# Triple versus dual antiplatelet therapy for coronary heart disease patients undergoing percutaneous coronary intervention: A meta-analysis

**DOI:** 10.3892/etm.2013.1238

**Published:** 2013-07-30

**Authors:** HONG ZHOU, XIAO-LING FENG, HONG-YING ZHANG, FEI-FEI XU, JIE ZHU

**Affiliations:** Department of Emergency, The Fourth Affiliated Hospital of China Medical University, Shenyang, Liaoning 110032, P.R. China

**Keywords:** cilostazol, antiplatelet therapy, percutaneous coronary intervention, coronary heart disease, meta-analysis

## Abstract

Coronary heart disease (CHD) is the leading cause of mortality worldwide. Previous studies have suggested that cilostazol-based triple antiplatelet therapy (TAT) may be more effective than conventional dual antiplatelet therapy (DAT) at improving the clinical outcomes of patients with CHD undergoing percutaneous coronary intervention (PCI). However, individually published results are inconclusive. The present meta-analysis evaluated controlled clinical studies to compare the clinical outcomes between TAT and DAT in patients with CHD undergoing PCI. Ten controlled clinical studies were included, with a total of 7,670 patients with CHD undergoing PCI. The total number included 3,925 patients treated with DAT (aspirin and clopidogrel) and 3745 patients treated with TAT (addition of cilostazol to DAT). The crude odds ratio (OR) with a 95% confidence interval (CI) was calculated with either the fixed or random effects model. The meta-analysis results indicated that patients in the TAT group had a significantly lower rate of restenosis compared with that of the DAT group (OR=0.59, 95% CI: 0.45–0.77; P<0.001). The rate of major adverse cardiac events (MACE) and target lesion revascularization (TLR) in the TAT group were significantly lower compared with those in the DAT group (MACE: OR=0.69, 95% CI: 0.56–0.85, P<0.001; TLR: OR=0.61, 95% CI: 0.43–0.88, P=0.008). However, no significant differences between the TAT and DAT groups in terms of mortality rate, myocardial infarction, target vessel revascularization and stent thrombosis were observed. In conclusion, the results of the present meta-analysis indicated that the efficacy and safety of cilostazol-based TAT therapy is greater than that of conventional DAT therapy for patients with CHD undergoing PCI.

## Introduction

Percutaneous coronary intervention (PCI), also known as coronary angioplasty, is a non-surgical method used to treat narrowed coronary arteries that supply the heart muscle with blood (1). PCI has been clinically applied for almost 30 years and has become one of the main treatments for coronary heart disease (CHD) (2). PCI with coronary stent implantation has been demonstrated to consistently reduce the symptoms of coronary artery disease and decrease cardiac ischemia; however, PCI has not been shown to reduce mortality rates in large clinical trials (3). The implantable vascular stents used during PCI procedures appear to increase the risk of coronary artery intimal injury and platelet activation, and may thereby increase the risk of thrombosis (4). This is significant, as the 1-year mortality rate of patients with myocardial infarction (MI) induced by thrombotic diseases is ∼15.8% (5). Therefore, antiplatelet therapy has become the focus of basic interventional cardiology studies and has received increased clinical attention in the last decade (6).

Dual antiplatelet therapy (DAT; aspirin and clopidogrel) is a mainstay of medical treatment following PCI, which reduces the risk of occurrence of stent thrombosis. However, studies have demonstrated that aspirin and clopidogrel resistance are occurring clinically with potentially severe consequences, including recurrent MI, stroke or mortality (7). Therefore, triple antiplatelet therapy (TAT; addition of cilostazol to DAT) has been increasingly studied (8). Cilostazol is a selective phosphodiesterase-3 inhibitor that is commonly used as a vasodilator with antiplatelet activity in patients with peripheral arterial disease (9). Adding cilostazol to aspirin and clopidogrel regimens may provide a more effective suppression of platelet P-selectin expression in patients with relatively high platelet activity (10). In addition, TAT has also been demonstrated to have anti-inflammatory, -apoptotic and -proliferative effects, as demonstrated by its reduction of intimal hyperplasia and restenosis following balloon angioplasty and stent implantation (11). Registry data have further identified that TAT reduces the rate of restenosis, incidence of clinical events and stent thrombosis, compared with DAT (12). However, controlled clinical studies that have examined the benefits of adding cilostazol to DAT in patients with CHD undergoing PCI with coronary stent implantation have obtained conflicted or inconclusive results. Therefore, the present meta-analysis aimed to compare differences in the clinical outcomes between DAT and TAT in patients with CHD undergoing PCI.

## Methods

### Literature search strategy

Relevant studies published prior to March 1, 2013, were identified by searches in Pubmed, Embase, Web of Science and Chinese BioMedical databases using the following terms: (‘coronary disease’ or ‘coronary diseases’ or ‘disease, coronary’ or ‘coronary heart disease’ or ‘heart disease, coronary’ or ‘stents’ or ‘stent’ or ‘drug-eluting stents’ or ‘bare metal stent’ or ‘percutaneous coronary intervention’, and ‘triple antiplatelet therapy’ or ‘dual antiplatelet therapy’ or ‘TAT’ or ‘DAT’ or ‘cilostazol’ or ‘clopidogrel’ or ‘aspirin’). References from eligible articles or textbooks were also reviewed to find further potential sources. Disagreements were resolved through discussion between authors.

### Inclusion and exclusion criteria

Studies included in the meta-analysis were required to meet the following criteria: i) controlled clinical study focusing on the differences in the clinical outcomes between TAT and DAT in patients with CHD undergoing PCI with coronary stent implantation; ii) patients in the DAT group were treated with aspirin and clopidogrel and patients in the TAT group were treated with cilostazol in addition to aspirin and clopidogrel; iii) the follow-up period was >1 month and iv) published data of the clinical outcomes were sufficient. Studies were excluded from the meta-analysis when they were: i) not controlled clinical studies relevant to TAT and DAT in patients with CHD undergoing PCI with coronary stent implantation; ii) duplicates of previous publications; iii) based on incomplete data; or iv) meta-analyses, letters, reviews or editorial articles. When more than one study by the same author and using the same case series was published, either the study with the largest sample size or the most recently published study was included.

### Data extraction

Using a standardized form, data from published studies were extracted independently by two authors. For each study, the following characteristics and numbers were collected: the authors, year of publication, country, ethnicity, study design, number of subjects, follow-up period, antiplatelet drug and dose, rate of restenosis and clinical events [major adverse cardiac events (MACE) and stent thrombosis]. MACE included mortality rate, MI, target lesion revascularization (TLR) and target vessel revascularization (TVR). In cases of conflicting evaluations, two authors discussed the issue in order to meet a consensus; if no agreement was reached, a third author decided.

### Statistical analysis

The crude odds ratio (OR) with 95% confidence interval (CI) was calculated. The statistical significance of the pooled OR was examined by the Z-test. Interstudy variations and heterogeneities were estimated using Cochran’s Q-statistic with P<0.05 indicating a statistically significant heterogeneity (13,14). The present meta-analysis also quantified the effect of heterogeneity by using the I^2^ index (range, 0–100%), which represents the proportion of interstudy variability attributed to heterogeneity, rather than to chance. When a significant Q-test (P<0.05) or an I^2^ index of >50% was obtained, indicating that heterogeneity existed among studies, the random effects model using the DerSimonian and Laird method (15) was conducted. However, when the Q-test was not significant (P>0.05) or the I^2^ index was <50%, the fixed effects model using the Mantel-Haenszel method (16) was used. To explore sources of heterogeneity, subgroup analysis was performed by follow-up periods. A sensitivity analysis was performed by omitting each study in turn, to assess the quality and consistency of the results. Begg’s funnel plot was used to detect publication bias. Egger’s linear regression test, which measures funnel plot asymmetry using a natural logarithm scale of OR, was also used to evaluate publication bias (17). To ensure the reliability and accuracy of the results, two authors examined the data independently using statistical software programs and obtained identical results. The P-values were two-sided and P<0.05 was considered to indicate a statistically significant difference. All analyses were calculated using Stata software, version 12.0 (StataCorp LP, College Station, TX, USA).

## Results

### Characteristics of the included studies

**Figure 1.**
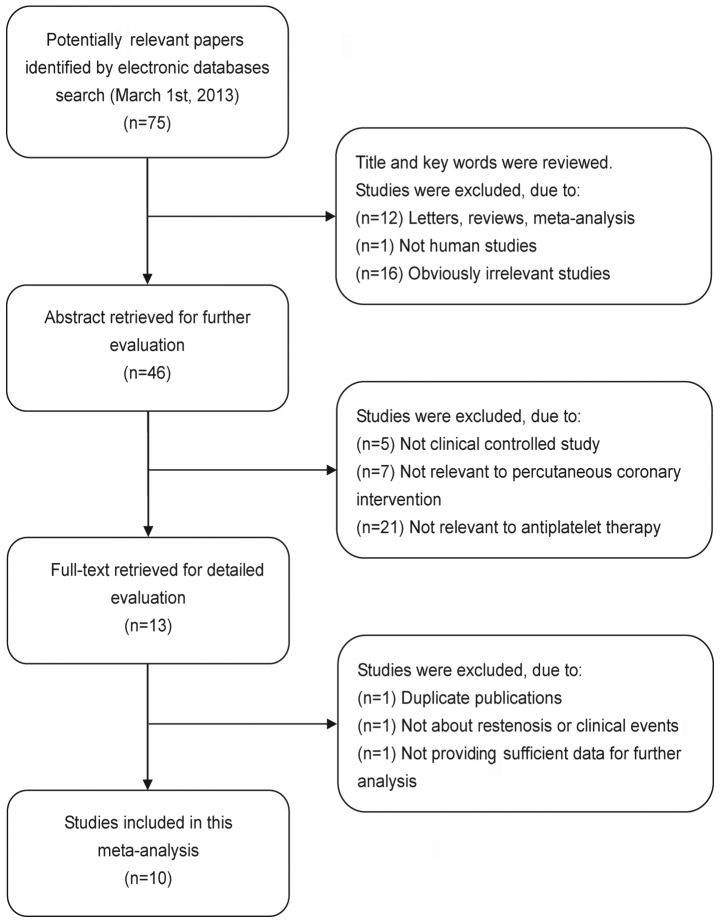
Flow chart of literature search and study selection.

In the present meta-analysis, according to the inclusion criteria, 10 controlled clinical studies were included (8,12,18–25) and 65 were excluded. The publication year of the included studies ranged from 2005 to 2011. The flow chart of study selection is shown in Fig. 1. A total of 7,670 patients with CHD undergoing PCI were involved in the present meta-analysis, including 3,925 patients treated with DAT (aspirin and clopidogrel) and 3,745 patients treated with TAT (addition of cilostazol to DAT). The doses of aspirin ranged from 100 to 200 mg/day, those of cilostazol ranged from 100 to 200 mg/day and the dose of clopidogrel was 75 mg/day. The main characteristics of the eligible studies are listed in Table I.

### Quantitative data synthesis

**Figure 2.**
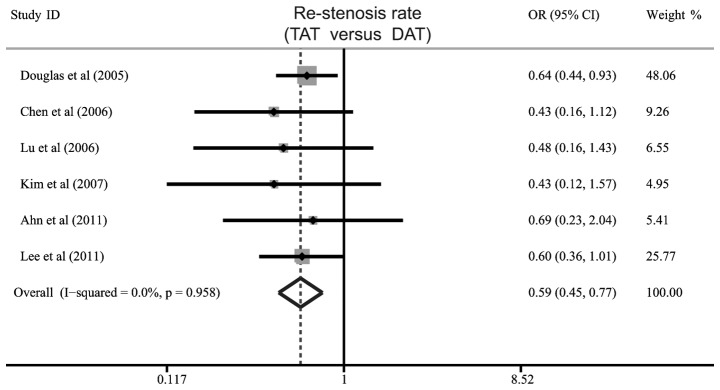
Forest plot of crude OR for differences in the restenosis rate between TAT and DAT. OR, odds ratio; TAT, triple antiplatelet therapy; DAT, dual antiplatelet therapy; CI, confidence interval.

Six studies identified a difference in the rate of restenosis between TAT and DAT for patients with CHD undergoing PCI. The heterogeneity was not significant (P=0.958, I^2^=0%), therefore, the fixed effects model was used. The meta-analysis results showed that patients in the TAT group had a significantly lower rate of restenosis than those in the DAT group (OR=0.59, 95% CI: 0.45–0.77, P<0.001; Fig. 2).

**Figure 3.**
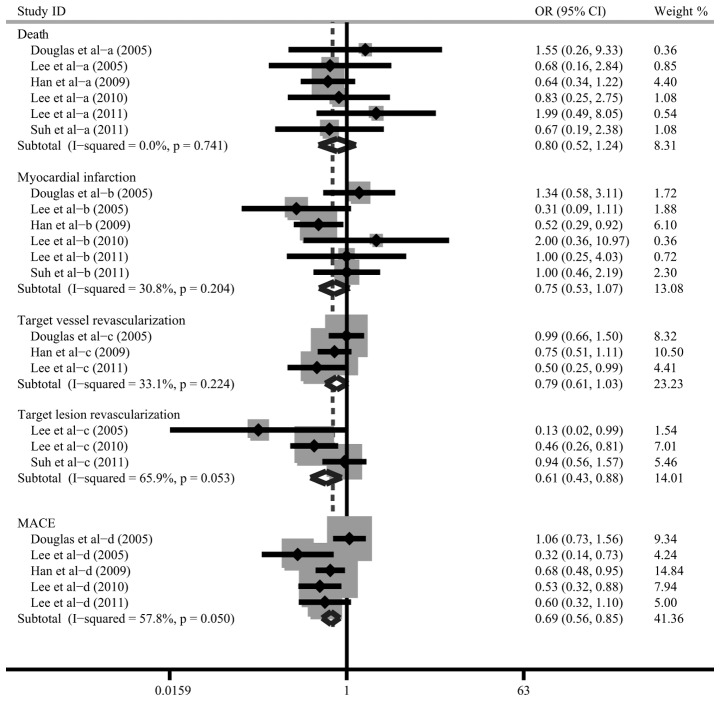
Forest plot of crude OR for differences in the rates of MACE between TAT and DAT. MACE, major adverse cardiac events; CI, confidence interval; OR, odds ratio; TAT, triple antiplatelet therapy; DAT, dual antiplatelet therapy.

**Figure 4.**
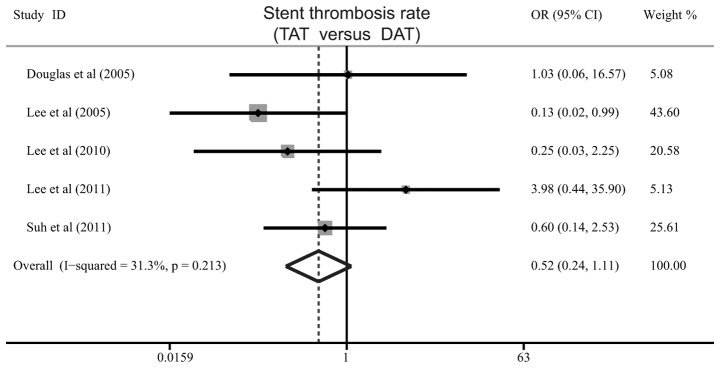
Forest plot of crude OR for differences in the rate of stent thrombosis between TAT and DAT. OR, odds ratio; TAT, triple antiplatelet therapy; DAT, dual antiplatelet therapy; CI, confidence interval.

The differences in the clinical events between TAT and DAT were compared in six studies. As no heterogeneity was identified, the fixed effects model was used. The meta-analysis results indicated that the rates of MACE and TLR in the TAT group were significantly lower compared with those in the DAT group (MACE: OR=0.69, 95% CI: 0.56–0.85, P<0.001; TLR: OR=0.61, 95% CI: 0.43–0.88, P=0.008). However, no significant differences were indicated in mortality rates (OR=0.80, 95% CI: 0.52–1.24, P=0.319), MI (OR=0.75, 95% CI: 0.53–1.07, P=0.109) and TVR (OR=0.79, 95% CI: 0.61–1.03, P=0.077) between the TAT and DAT groups (Fig. 3). Additionally, no significant difference was identified in the rate of stent thrombosis between the TAT and DAT groups (OR=0.52, 95% CI: 0.24–1.11, P=0.091; Fig. 4).

**Figure 5.**
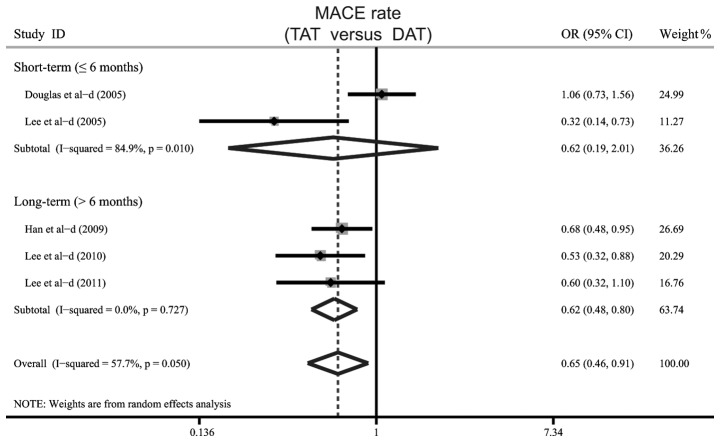
Subgroup analysis by follow-up periods of crude OR for the rate of MACE between TAT and DAT. OR, odds ratio; MACE, major adverse cardia events; TAT, triple antiplatelet therapy; DAT, dual antiplatelet therapy; CI, confidence interval.

When examining the potential factors that may have impacted the results, a further subgroup analysis was performed based on the follow-up periods. The results indicated a significant difference in the rate of MACE between TAT and DAT in the long-term (>6 months) follow-up subgroups (OR=0.62, 95% CI: 0.48–0.80, P<0.001). However, no significant difference in the rate of MACE was indicated in the short-term (≤6 months) follow-up subgroups (OR=0.62, 95% CI: 0.19–2.01, P=0.421; Fig. 5).

### Sensitivity analysis and publication bias

**Figure 6.**
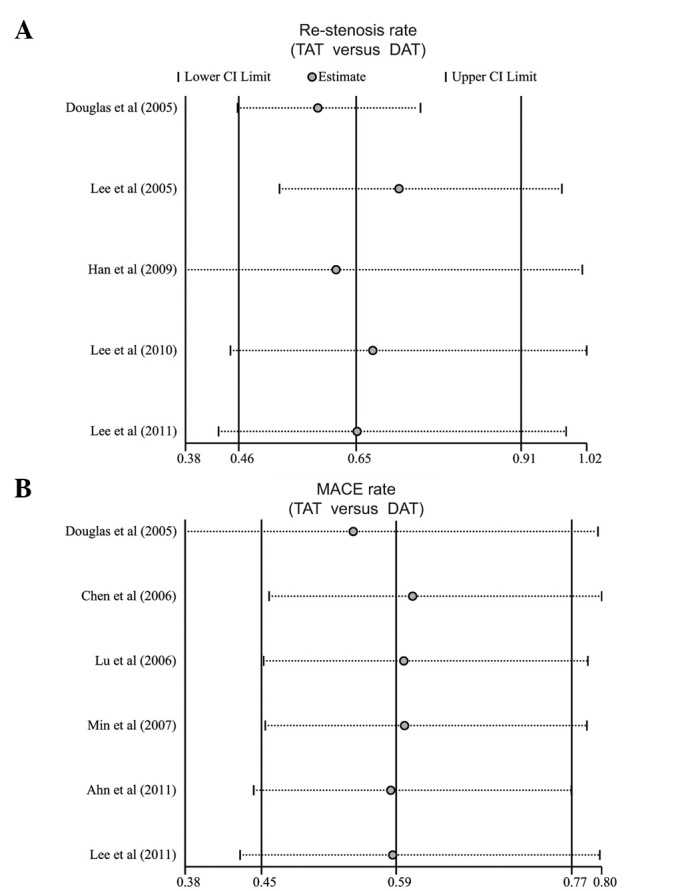
Sensitivity analysis of the summary odds ratio coefficients on the rates of restenosis and MACE between TAT and DAT. (A) Restenosis rate; (B) MACE rate. Results were computed by omitting each study in turn. Meta-analysis random-effects estimates (exponential form) were used. The two ends of the dotted lines represent the 95% CI. MACE, major adverse cardiac events; TAT, triple antiplatelet therapy; DAT, dual antiplatelet therpay; CI, confidence interval.

Sensitivity analysis was performed to assess the influence of each individual study on the pooled ORs by omitting individual studies. Analysis of the results suggested that no individual study significantly affected the pooled ORs of the rate of restenosis and MACE (Fig. 6), indicating a statistically robust result.

**Figure 7.**
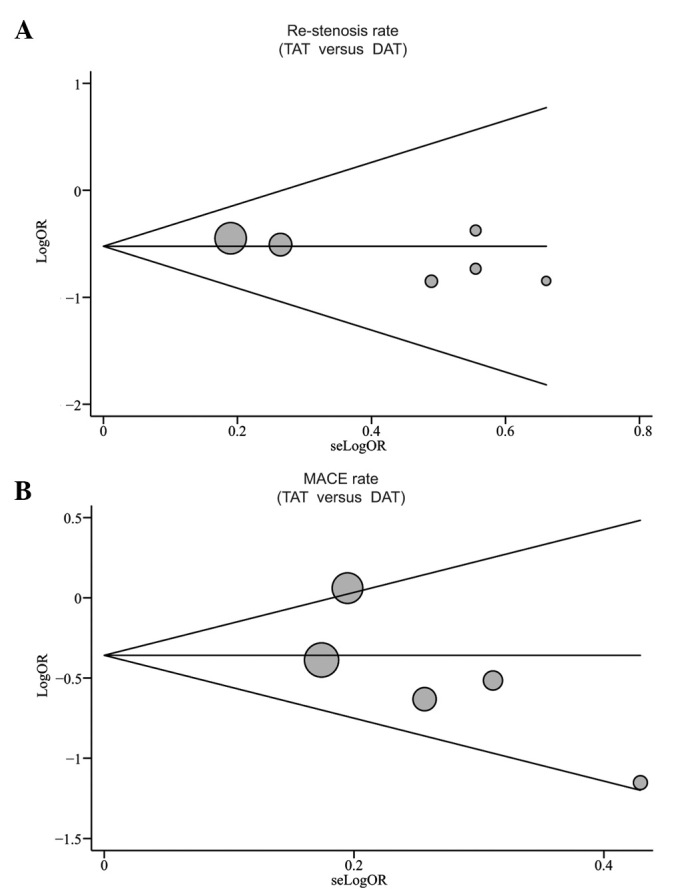
Begg’s funnel plot of publication bias in a selection of studies on the rates of restenosis and MACE between TAT and DAT. (A) Restenosis rate; (B) MACE rate. Each point represents a separate study for the indicated association. Log (OR), natural logarithm of OR. Horizontal line, mean magnitude of the effect. MACE, major adverse cardiac events; TAT, triple antiplatelet therapy; DAT, dual antiplatelet therpay; OR, odds ratio.

Publication bias exists to the extent that the available research results are not representative of all research results. Begg’s funnel plot and Egger’s linear regression test were performed to assess the publication bias of the included studies. The shapes of the funnel plots did not demonstrate any obvious asymmetry (Fig. 7). Egger’s test also showed that there was no statistical indication of publication bias for the rates of restenosis and MACE (restenosis: t=−2.04, P=0.111; MACE: t=−1.76, P=0.177).

## Discussion

The clinical application of PCI with coronary stent implantation has markedly increased, which has significantly improved the outcomes in patients with CHD (26). However, serious complications following implantation of coronary stents remain a significant clinical problem and may result in failure of PCI to provide long-term benefits. Over the past decade, numerous studies have indicated that the use of adjunctive antiplatelet agents has resulted in decreased stent-associated thrombosis and is important in improving the final outcomes of PCI (27). Two meta-analyses by Tamhane *et al* (11) and Singh *et al* (28) also indicated that patients treated with TAT exhibited a lower restenosis rate than those treated with DAT (11,28). However, these meta-analyses did not provide reliable results demonstrating the differences in the clinical outcomes between TAT and DAT for patients with CHD undergoing PCI with coronary stent implantation. Therefore, in view of the conflicting results from previous studies and the insufficient statistical power of the two previous meta-analyses, the present meta-analysis was performed in order to update previous meta-analyses and provide a comprehensive conclusion.

In the present meta-analysis, 10 clinical studies were included with a total of 7,670 patients with CHD undergoing PCI with coronary stent implantation, including 3,745 patients in the TAT group and 3,925 patients in the DAT group. The predominant finding of this meta-analysis was that the rates of restenosis, MACE and TLR in the TAT group were significantly lower compared with those of the DAT group. Furthermore, when the follow-up periods were stratified into short-term (≤6 months) and long-term (>6 months) subgroups, it was noteworthy that a significant difference in the rate of MACE between TAT and DAT was only observed in the long-term follow-up subgroup. The substantial improvement due to the addition of cilostazol to DAT identified by the current meta-analysis is consistent with the findings of several previous studies. This is due to cilostazol inhibiting the progression of carotid intima-media thickness and thus producing an additional anti-proliferative effect, regardless of the fact that DAT itself is a potent therapy (23,29–31). Notably, the significantly reduced rate of MACE in the long-term follow-up subgroup is inconsistent with the findings of two previous meta-analyses, which indicated that there was no difference in the clinical outcomes of MACE between DAT and TAT treatment groups (11,28). A potential reason for the discrepancy may be the limited number of included studies. The controversial results may also be caused by the high degree of heterogeneity in the different follow-up periods. In the present meta-analysis, no significant differences in the rates of mortality, MI and TVR were observed between TAT and DAT for patients with CHD undergoing PCI. Additionally, no significant difference in the rate of stent thrombosis between TAT and DAT was determined, which is in accordance with a previous meta-analysis (32). These results suggest that TAT may be no different from DAT in terms of its effectiveness as an adjunctive therapy to coronary stents and as a preventative measure against stent-associated thrombosis. This is regardless of the different antiplatelet mechanisms of TAT, which may result in suppression of platelet aggregation (32). The precise effect of adding cilostazol to DAT on the incidence rates of various clinical events requires further study before conclusions regarding these outcomes may be made.

Similar to previous meta-analyses, the present meta-analysis also has certain limitations. The sample size was relatively small and may not have provided sufficient statistical power to estimate the differences in the clinical outcomes between TAT and DAT for patients with CHD undergoing PCI. A meta-analysis, as a type of retrospective study, may encounter recall or selection bias, which may influence the reliability of the results. A further limitation, was that each pretreatment regimen was not consistent and the doses of aspirin or cilostazol were variable. Moreover, the type of stent used in each patient was not consistent and there may have been differences with regard to the effectiveness of the antiplatelet agents. Although all participants in each study were required to meet similar inclusion criteria, there may have been potential factors that were not taken into account that may have influenced the results. Therefore, the results of this meta-analysis should be interpreted with caution, owing to the potential heterogeneity among trials.

In conclusion, the present meta-analysis suggests that the efficacy and safety of cilostazol-based TAT may be greater than that of conventional DAT for treating patients with CHD undergoing PCI. However, additional well-designed and randomized controlled trials are required to further investigate and clarify the differences in the clinical outcomes between TAT and DAT for patients with CHD.

## Figures and Tables

**Table I.** Characteristics of studies included in this meta-analysis.

Author	Year	Country	Ethnicity			Follow-up (months)	Drug doses					Clinical outcomes

				Number			TAT			DAT		
		
				TAT	DAT		Aspirin	Clopidogrel	Cilostazol	Aspirin	Clopidogrel	
Douglas *et al*	2005	USA	Caucasian	354	351	6	NR	75 mg/day	100 mg/bid	NR	75 mg/day	Clinical events Restenosis
Lee *et al*	2005	Korea	Asian	1415	1597	1	200 mg/day	75/300 mg/day	100/200 mg/bid	200 mg/day	75/300 mg/day	Clinical events
Chen *et al*	2006	China	Asian	60	60	6	100 mg/day	75 mg/day	200 mg/day	100 mg/day	75 mg/day	Restenosis
Lu *et al*	2006	China	Asian	60	58	6	100 mg/day	75 mg/day	100 mg/bid	100 mg/day	75 mg/day	Restenosis
Kim *et al*	2007	Korea	Asian	31	28	6	100 mg/day	75 mg/day	100 mg/bid	100 mg/day	75 mg/day	Restenosis
Han *et al*	2009	Korea	Asian	604	608	12	300 mg/day; 100 mg/day after 1 month	75 mg/day	100 mg/bid	300 mg/day; 100 mg/day after 1 month	75 mg/day	Clinical events
Lee *et al*	2010	Korea	Asian	450	450	24	200 mg/day	75 mg/day	200 mg/day	200 mg/day	75 mg/day	Clinical events
Ahn *et al*	2011	Korea	Asian	64	66	8	100 mg/day	75 mg/day	200 mg/day	100 mg/day	75 mg/day	Restenosis
Lee *et al*	2011	Korea	Asian	250	249	8	200 mg/day	75 mg/day	200 mg/day	200 mg/day	75 mg/day	Clinical events Restenosis
Suh *et al*	2011	Korea	Asian	457	458	6	100 mg/day	75 mg/day	200 mg/day	100 mg/day	75 mg/day	Clinical events

TAT, triple antiplatelet therapy; DAT, dual antiplatelet therapy; NR, not reported.
